# A comparison of case definitions for infant atopic dermatitis in a multicenter prospective cohort study

**DOI:** 10.1002/hsr2.324

**Published:** 2021-07-12

**Authors:** David X. Zheng, Ruth J. Geller, Lacey B. Robinson, Markus D. Boos, Carlos A. Camargo

**Affiliations:** ^1^ Department of Dermatology Case Western Reserve University School of Medicine Cleveland Ohio USA; ^2^ Department of Emergency Medicine Massachusetts General Hospital, Harvard Medical School Boston Massachusetts USA; ^3^ Division of Rheumatology Allergy and Immunology, Massachusetts General Hospital, Harvard Medical School Boston Massachusetts USA; ^4^ Division of Dermatology Seattle Children's Hospital Seattle Washington USA

## Abstract

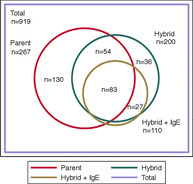

This study identified two infant AD case definitions that were strongly associated with known AD risk factors. These case definitions can be used to study novel AD risk factors in large cohort studies, potentially providing new insights into the epidemiology of infant AD.

## 1 BACKGROUND

Atopic dermatitis (AD) is an inflammatory skin disease characterized by dryness, pruritus, and a chronically relapsing course often beginning in infancy.^1^ Given the inverse association between age of AD onset and risk of developing food allergy, allergic rhinitis, and asthma,^2^, ^3^ understanding infant AD epidemiology may inform research on this “atopic march.”^4^

Epidemiologic definitions of AD vary across cohort studies due to the heterogeneous and episodic nature of AD.^5^ While examination by a board‐certified dermatologist is ideal for diagnosis, this usually is not feasible in large cohorts. The International Study of Asthma and Allergies in Children questionnaire and the Hanifin and Rajka criteria are two validated diagnostic instruments used in research and clinical settings.^6^, ^7^ Yet, these approaches remain limited because they were not designed for diagnosing infant AD, nor do they incorporate medical records or more objective markers of atopy, such as immunoglobulin E (IgE). Thus, there is a need for infant AD case definitions based on components more readily available in epidemiologic studies.

To address this knowledge gap, we analyzed data from a large cohort of infants.^8^ We combined data from parent report, medical record review, and laboratory testing to establish a panel of case definitions for infant AD. We compared the associations of these case definitions with *known* infant AD risk factors.^1^, ^9^ Our rationale was that the case definition(s) most strongly associated with known risk factors would best capture infant AD.

## 2 METHODS

### 2.1 Cohort selection

We conducted a planned secondary analysis of the 35th Multicenter Airway Research Collaboration (MARC‐35), a multicenter, prospective cohort study of infants (age < 1 year) hospitalized for bronchiolitis. Enrollment was conducted at 17 hospitals across 14 US states during 2011 to 2014 winter seasons (Data S1).^8^ The institutional review board at each hospital approved the study, and the written informed consent was obtained from parents/guardians.

### 2.2 Data collection

At enrollment, investigators conducted a structured interview with parents/guardians to assess patients' demographic characteristics, family and medical history, and clinical details; blood specimens were collected. After the bronchiolitis hospitalization, study staff interviewed parents/guardians by telephone at 6‐month intervals, in addition to medical record review by trained physicians.

### 2.3 Primary exposures and outcome measures

The exposures were known predictors of infant AD, collected by parent report at enrollment: maternal history of AD, paternal history of AD, either parent with history of AD, maternal history of allergic rhinitis, and maternal history of asthma.^1^, ^9^

The outcome measures were a panel of case definitions for infant AD. During both enrollment and the age 12‐month interview, parents/guardians were asked if the infant had a history of eczema/AD, defined as an “itchy, scaly rash that comes and goes.”^6^ We defined parent‐reported AD as an affirmative response at either interview. Following a standardized protocol, physicians reviewed medical records to assess whether there was documentation of clinician‐diagnosed AD (including “eczema,” “atopic eczema”; excluding “diaper rash,” “seborrheic dermatitis/cradle cap,” “contact dermatitis”), documentation of no AD, or lack of AD‐related documentation. Additionally, physicians reviewed parent‐reported data in combination with medical record data (“hybrid approach”) to classify infants as having AD, no AD, or lack of AD‐related documentation. Serum total IgE (tIgE) and specific IgE (sIgE) levels were measured at Phadia Immunology Reference Laboratory (Portage, Michigan) (Data S1).^10^

We compiled the following case definitions: (a) parent‐reported AD from interviews; (b) clinician‐diagnosed AD from medical records; (c) hybrid approach (physician assessment of AD status, based on parent‐reported AD and clinician‐diagnosed AD); (d) hybrid plus elevated tIgE; (e) hybrid plus elevated sIgE; and (f) hybrid plus elevated tIgE or sIgE.

### 2.4 Statistical analysis

Analyses were performed using Stata 14.2 (Stata Corp, College Station, Texas). We calculated descriptive statistics using percentages and medians with interquartile ranges (IQR), overall and by AD definition. We assessed associations of AD risk factors with AD case definitions using log‐binomial models to calculate risk ratios (RRs) and 95% confidence intervals (95% CIs). Regression models used a clustered sandwich estimator to account for potential clustering by site. Complete case analysis was used for missing data.

## 3 RESULTS

The study enrolled 1016 infants, of whom 921 (91%) were followed longitudinally (“analytic cohort”); 95 (9%) contributed enrollment data only (“nonanalytic cohort”). We excluded two participants who died during infancy, one from the analytic and one from the nonanalytic cohort. The analytic and nonanalytic cohorts did not significantly differ in demographics (Table S1).

**FIGURE 1:**
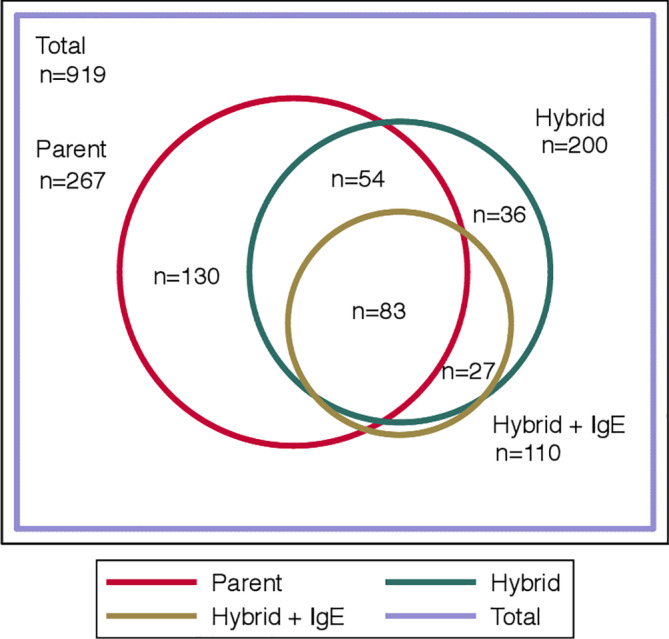
Overlap between three definitions of infant atopic dermatitis (AD). The three definitions shown are parent‐reported AD, physician‐ascertained AD based on parent report and medical record review (“hybrid AD”) and hybrid AD with elevated total immunoglobulin E (tIgE) or specific immunoglobulin E (sIgE). Elevated sIgE includes positive results to any food allergen using ImmunoCAP or positive results to any food or aeroallergen using Immuno Solid‐phase Allergen Chip (ISAC)

In the analytic cohort, the median age was 3 months (IQR 2‐6), 60% were male, and 43% were non‐Hispanic white. AD prevalence ranged from 55/919 (6%) for hybrid plus elevated sIgE to 268/920 (29%) for parent‐reported AD (Table S2). Among 267 infants with parent‐reported AD and complete medical record data, 137 (51%) also had hybrid AD (Figure S1). Among 200 infants with hybrid AD, 159/200 (80%) had elevated tIgE or sIgE (Figure S2) and 83/200 (42%) had parent‐reported AD and hybrid AD plus elevated tIgE or sIgE (Figure 1).

In regression models, the strongest associations of AD risk factors were observed with parent‐reported infant AD (Table 1). For example, maternal history of AD had the strongest association with parent‐reported infant AD (RR 2.05 [95% CI 1.51, 2.79]), followed by hybrid AD plus elevated tIgE or sIgE (RR 1.99 [95% CI 1.33, 2.99]).

**TABLE 1:** Associations between major atopic dermatitis risk factors and six definitions of infant atopic dermatitis

	AD definition					
	Parent‐reported AD	Clinician‐diagnosed AD	Hybrid AD^a^	Hybrid AD + elevated tIgE	Hybrid AD + elevated sIgE^b^	Hybrid AD + elevated tIgE or sIgE
Exposure variables	*Risk ratio (95% confidence interval)*					
Maternal AD history						
Yes	2.05 (1.51, 2.79)	1.29 (0.91, 1.82)	1.40 (1.01, 1.93)	1.92 (1.29, 2.86)	1.69 (0.96, 2.98)	1.99 (1.33, 2.99)
No	1.00 (Reference)	1.00 (Reference)	1.00 (Reference)	1.00 (Reference)	1.00 (Reference)	1.00 (Reference)
Paternal AD history^c^						
Yes	2.20 (1.66, 2.91)	1.70 (1.14, 2.53)	1.81 (1.28, 2.54)	1.77 (0.93, 3.37)	2.10 (1.31, 3.37)	1.80 (1.05, 3.09)
No	1.00 (Reference)	1.00 (Reference)	1.00 (Reference)	1.00 (Reference)	1.00 (Reference)	1.00 (Reference)
Maternal or paternal AD history^c^						
Yes	2.22 (1.69, 2.93)	1.49 (1.05, 2.11)	1.64 (1.18, 2.28)	2.02 (1.25, 3.29)	1.82 (1.07, 3.11)	2.02 (1.27, 3.22)
No	1.00 (Reference)	1.00 (Reference)	1.00 (Reference)	1.00 (Reference)	1.00 (Reference)	1.00 (Reference)
Maternal allergic rhinitis history						
Yes	1.65 (1.24, 2.20)	1.06 (0.70, 1.61)	1.11 (0.71, 1.74)	0.99 (0.54, 1.83)	1.24 (0.64, 2.40)	1.03 (0.57, 1.83)
No	1.00 (Reference)	1.00 (Reference)	1.00 (Reference)	1.00 (Reference)	1.00 (Reference)	1.00 (Reference)
Maternal asthma history						
Yes	1.66 (1.25, 2.20)	1.34 (1.07, 1.68)	1.40 (1.12, 1.74)	1.56 (1.16, 2.10)	1.78 (1.17, 2.71)	1.57 (1.16, 2.13)
No	1.00 (Reference)	1.00 (Reference)	1.00 (Reference)	1.00 (Reference)	1.00 (Reference)	1.00 (Reference)

Abbreviations: AD, atopic dermatitis; sIgE, specific IgE; tIgE, total IgE.

^a^ Physician‐ascertained AD based on parent report and medical record review.

^b^ Positive results to any food allergen using ImmunoCAP or positive results to any food or aeroallergen using ISAC.

^c^ Results for the missing data category are not shown.

## 4 DISCUSSION

The prevalence of infant AD ranged from 6% to 29% across definitions, encompassing AD prevalence estimates from previous cohort studies of healthy Polish (17%)^11^ and Australian (20%)^12^ infants, and infants with severe bronchiolitis (14%).^13^ Prevalence of parent‐reported AD and hybrid approach plus elevated tIgE or sIgE was 29% and 12%, respectively. The observed difference in prevalence between the two case definitions most strongly associated with known infant AD risk factors may be explained by the heterogeneous nature of AD.^1^ Regardless, we acknowledge the possibility of greater false positives when using a less specific case definition (eg, parent‐reported AD).

Although clinical examination by a dermatologist remains the gold standard for AD diagnosis,^1^ the validity of caregiver‐reported AD has previously been established. Silverberg et al found caregiver‐reported history of childhood AD to have high sensitivity (70%), specificity (96%), and positive predictive value (87%) when compared with a dermatologist's diagnosis of AD using Hanifin and Rajka's criteria.^14^ Furthermore, the validity of self‐reported atopic disease (the basis of our primary exposures) has been established in other large cohort studies.^14^, ^15^

Dharma et al, however, reported that questionnaire data cannot accurately substitute for assessment by healthcare professionals using validated criteria in diagnosing infant AD.^16^ Clinical examination by a dermatologist is usually not feasible in large epidemiologic cohorts. Despite this limitation, several of our case definitions incorporated clinician‐diagnosed AD, obtained from medical record review. Our hybrid approach adds another layer of diagnostic validation by incorporating a trained physician chart reviewer's best judgment of AD status based on synthesis of all available information from parent report and medical record review.

This study has limitations, most notably the lack of validation against clinical examination by a dermatologist. Moreover, our sample consisted of infants with severe bronchiolitis. Although bronchiolitis is the most common reason for hospitalization of US infants,^17^ our findings may be less generalizable to other infant populations.

## 5 CONCLUSIONS

We compared potential case definitions for infant AD and propose the use of: (a) parent‐reported AD and (b) hybrid approach (parent‐reported and clinician‐diagnosed AD) plus elevated tIgE or sIgE. These results can inform future epidemiologic studies of novel risk factors for infant AD and the atopic march.

## FUNDING

Mr. Zheng was supported by the Case Western Reserve University School of Medicine Dean's Award (Cleveland, Ohio). This study was supported by the grants U01 AI‐087881, R01 AI‐114552, and UG3 OD‐023253 from the National Institutes of Health (Bethesda, Maryland). The content of this manuscript is solely the responsibility of the authors and does not necessarily represent the official views of the National Institutes of Health. The supporting sources had no involvement in the study design; collection, analysis, and interpretation of data; writing of the report; or the decision to submit the report for publication.

## CONFLICT OF INTEREST

The authors report no conflicts of interest relevant to this work.

## AUTHOR CONTRIBUTIONS

Conceptualization: David X. Zheng, Ruth J. Geller, Carlos A. Camargo Jr.

Data curation: Ruth J. Geller.

Formal analysis: Ruth J. Geller.

Funding Acquisition: Carlos A. Camargo Jr.

Investigation: Lacey B. Robinson, Markus D. Boos.

Methodology: David X. Zheng, Ruth J. Geller, Carlos A. Camargo Jr.

Project administration: Markus D. Boos.

Supervision: Carlos A. Camargo Jr.

Validation: Lacey B. Robinson.

Writing–Original Draft: David X. Zheng.

Writing–Review and Editing: Ruth J. Geller, Lacey B. Robinson, Markus D. Boos, Carlos A. Camargo Jr.

All authors have read and approved the final version of the manuscript.

Carlos A. Camargo Jr. had full access to all of the data in this study and takes complete responsibility for the integrity of the data and the accuracy of the data analysis.

## TRANSPARENCY STATEMENT

David X. Zheng affirms that this manuscript is an honest, accurate, and transparent account of the study being reported; that no important aspects of the study have been omitted; and that any discrepancies from the study as planned have been explained.

## Supporting information


**Data S1.** Supporting Information.

**Figure S1.** Overlap between three definitions of infant atopic dermatitis
The three definitions shown are parent‐reported atopic dermatitis, clinician‐diagnosed atopic dermatitis ascertained from medical record review and physician‐ascertained atopic dermatitis based on parent report and medical record review (“hybrid” definition).

**Figure S2.** Overlap between elevated total IgE (tIgE) and specific IgE (sIgE) among infants with atopic dermatitis (“hybrid” definition)
Elevated sIgE includes positive results to any food allergen using ImmunoCAP or positive results to any food or aeroallergen using Immuno Solid‐phase Allergen Chip. “Hybrid” atopic dermatitis refers to physician‐ascertained atopic dermatitis based on parent report and medical record review. IgE, immunoglobulin E

## Data Availability

The authors confirm that the data supporting the findings of this study are available within the article and its supplementary materials.
