# Supplementary material for: A comparison of case definitions for infant atopic dermatitis in a multicenter prospective cohort study
Source: Health Sci Rep. 2021 Jul 12;4(3):e324. doi: 10.1002/hsr2.324 (PMC8273877; doi:10.1002/hsr2.324)
Supplement: Supplementary file 1 — Data S1. Supporting Information. [file HSR2-4-e324-s001.docx]

**SUPPLEMENT**

**A comparison of case definitions for infant atopic dermatitis in a multicenter prospective cohort study**

David X. Zheng, BA;^1,2^ Ruth J. Geller, MHS;^2^ Lacey B. Robinson, MD;^3^ Markus D. Boos, MD, PhD;^4^ Carlos A. Camargo Jr., MD, DrPH^2^

^1^ Department of Dermatology, Case Western Reserve University School of Medicine, Cleveland

^2^ Department of Emergency Medicine, Massachusetts General Hospital, Harvard Medical School, Boston

^3^ Division of Rheumatology, Allergy and Immunology, Massachusetts General Hospital, Harvard Medical School, Boston

^4^ Division of Dermatology, Seattle Children’s Hospital, Seattle

**Address manuscript correspondence to:** Carlos A. Camargo Jr., MD, DrPH

Massachusetts General Hospital, 125 Nashua St., Suite 920, Boston, MA, 02114

Phone: (617) 726-5276. Fax: (617) 724-4050. Email: [ccamargo@partners.org](mailto:ccamargo@partners.org)

**Table of Contents**

- Appendix p. 2
- Supplementary Methods p. 3
- Supplementary Table 1 p. 4-5
- Supplementary Table 2 p. 6-7
- Supplementary Figure 1 p. 8
- Supplementary Figure 2 p. 9

**Appendix.** Principal investigators at the 17 participating sites in MARC-35

| Amy D. Thompson, MD | Alfred I. duPont Hospital for Children, Wilmington, DE |
| --- | --- |
| Federico R. Laham, MD, MS | Arnold Palmer Hospital for Children, Orlando, FL |
| Jonathan M. Mansbach, MD, MPH | Boston Children's Hospital, Boston, MA |
| Vincent J. Wang, MD, MHA; and Susan Wu, MD | Children's Hospital of Los Angeles, Los Angeles, CA |
| Michelle B. Dunn, MD; and Jonathan M. Spergel, MD, PhD | Children's Hospital of Philadelphia, Philadelphia, PA |
| Juan C. Celedón, MD, DrPH | Children's Hospital of Pittsburgh, Pittsburgh, PA |
| Michael R. Gomez, MD, MS-HCA; and Nancy R. Inhofe, MD | Children's Hospital at St. Francis, Tulsa, OK |
| Brian M. Pate, MD; and Henry T. Puls, MD | Children's Mercy Hospital & Clinics, Kansas City, MO |
| Stephen J. Teach, MD, MPH | Children's National Medical Center, Washington, DC |
| Stephen C. Porter, MD, MSc, MPH; and Richard T. Strait, MD | Cincinnati Children's Hospital and Medical Center, Cincinnati, OH |
| Ilana Y. Waynik, MD | Connecticut Children's Medical Center, Hartford, CT |
| Sujit S. Iyer, MD | Dell Children's Medical Center of Central Texas, Austin, TX |
| Ari R. Cohen, MD; Margaret Samuels-Kalow, MD; and Wayne G. Shreffler, MD, PhD | Massachusetts General Hospital, Boston, MA |
| Michelle D. Stevenson, MD, MS | Norton Children’s Hospital and the University of Louisville, Louisville, KY |
| Cindy S. Bauer, MD; and Anne K. Beasley, MD | Phoenix Children's Hospital, Phoenix, AZ |
| Markus Boos, MD, PhD; and Thida Ong, MD | Seattle Children's Hospital, Seattle, WA |
| Charles G. Macias, MD, MPH; and Sarah Meskill, MD | Texas Children's Hospital, Houston, TX |

**Supplementary Methods.** Serum total and specific IgE determination

Serum tIgE and sIgE concentrations were measured at Phadia Immunology Reference Laboratory (Portage, MI). Elevated tIgE was defined using age-specific cutoffs provided by Phadia as >2.3 kU/L (ages 0-6 weeks), >4.1 kU/L (ages 6.1 weeks - 3 months), >7.3 kU/L (ages 3.1-6 months), >10 kU/L (ages 6.1-9 months), and >13 kU/L (ages 9.1-11.9 months). We measured sIgE using two assays, ImmunoCAP and Immuno Solid-phase Allergen Chip (ISAC). We used ImmunoCAP to assess sensitization to cow’s milk, egg white, peanut, cashew nut, and walnut (sensitization defined as ≥0.35 kU/L). We used ISAC to assess sensitization to 19 food allergens and 10 aeroallergens (sensitization defined as ≥0.30 ISAC Standardized Units).

**Supplementary Table 1.** Cohort characteristics, overall and by analytic vs. non-analytic cohort

|  | **Overall**  (n=1014) | **Analytic**  **cohort**  (n=920) | **Non-analytic cohort**  (n=94) | **P-value^†^** |
| --- | --- | --- | --- | --- |
|  | *Median (IQR)* | | |  |
| *Child characteristics* |  |  |  |  |
| Age at enrollment, mo. | 3 (2-6) | 3 (2-6) | 3 (2-6) | 0.50 |
|  | *n (%)* | | |  |
| Sex |  |  |  | 0.78 |
| Male | 607 (60) | 552 (60) | 55 (59) |  |
| Female | 407 (40) | 368 (40) | 39 (41) |  |
| Race/ethnicity |  |  |  | 0.06 |
| Non-Hispanic White | 428 (42) | 400 (43) | 28 (30) |  |
| Non-Hispanic Black | 239 (24) | 210 (23) | 29 (31) |  |
| Hispanic | 308 (30) | 275 (30) | 33 (35) |  |
| Other | 39 (4) | 35 (4) | 4 (4) |  |
| Intensive care during enrollment hospitalization^‡^ | 162 (16) | 141 (15) | 21 (22) | 0.08 |
| *Exposure variables* |  |  |  |  |
| Maternal AD history |  |  |  | 0.04 |
| Yes | 136 (14) | 117 (13) | 19 (21) |  |
| No | 867 (86) | 794 (87) | 73 (79) |  |
| Paternal AD history |  |  |  | 0.30 |
| Yes | 81 (8) | 73 (8) | 8 (9) |  |
| No | 872 (86) | 795 (86) | 77 (82) |  |
| Unknown | 61 (6) | 52 (6) | 9 (10) |  |
| Maternal or paternal AD history |  |  |  | 0.31 |
| Yes | 198 (20) | 175 (19) | 23 (24) |  |
| No | 757 (75) | 693 (75) | 64 (68) |  |
| Unknown | 59 (6) | 52 (6) | 7 (7) |  |
| Maternal allergic rhinitis history |  |  |  | 0.71 |
| Yes | 235 (23) | 212 (23) | 23 (25) |  |
| No | 768 (77) | 699 (77) | 69 (75) |  |
| Maternal asthma history |  |  |  | 0.43 |
| Yes | 218 (22) | 195 (21) | 23 (25) |  |
| No | 785 (78) | 716 (79) | 69 (75) |  |
| *AD definition components* |  |  |  |  |
| Parent-reported AD at enrollment | 149 (15) | 137 (15) | 12 (13) | 0.58 |
| AD in medical record | 210 (21) | 195 (21) | 15 (16) | 0.23 |
| Hybrid AD^§^ | 215 (21) | 200 (22) | 15 (16) | 0.19 |
| Elevated tIgE | 432 (43) | 387 (42) | 45 (48) | 0.28 |
| Elevated sIgE^¶^ | 204 (20) | 182 (20) | 22 (23) | 0.40 |
| Elevated tIgE or sIgE | 462 (46) | 415 (45) | 47 (50) | 0.36 |

Abbreviations: AD, atopic dermatitis; IQR, interquartile range; sIgE, specific IgE; tIgE, total IgE

^†^ P-values are from chi-square, Fisher’s exact, and Kruskal-Wallis tests, as appropriate

^‡^ Intensive care unit, continuous positive airway pressure, and/or intubation

^§^ Physician-ascertained AD based on parent report and medical record review

^¶^ Positive results to any food allergen using ImmunoCAP, or positive results to any food or aeroallergen using ISAC

**Supplementary Table 2.** Descriptive cohort characteristics, overall and by atopic dermatitis definition

|  | AD definition | | | | | | |
| --- | --- | --- | --- | --- | --- | --- | --- |
|  | **Overall (n=920)** | **Parent-reported AD**  **(n=268)** | **Clinician-diagnosed AD (n=195)** | **Hybrid AD^†^**  **(n=200)** | **Hybrid AD + elevated tIgE (n=104)** | **Hybrid AD + elevated sIgE^‡^ (n=55)** | **Hybrid AD + elevated tIgE or sIgE (n=110)** |
|  | n (%) | n (%) | n (%) | n (%) | n (%) | n (%) | n (%) |
| *Child characteristics* |  |  |  |  |  |  |  |
| Age at enrollment (median [IQR]), mo. | 3 (2-6) | 4 (2-7) | 3 (2-6) | 3 (2-6) | 4 (2-6) | 4 (2-7) | 4 (2-6) |
| Sex |  |  |  |  |  |  |  |
| Male | 552 (60) | 166 (62) | 134 (69) | 137 (69) | 65 (63) | 33 (60) | 69 (63) |
| Female | 368 (40) | 102 (38) | 61 (31) | 63 (32) | 39 (38) | 22 (40) | 41 (37) |
| Race/ethnicity |  |  |  |  |  |  |  |
| Non-Hispanic White | 400 (43) | 116 (43) | 73 (37) | 74 (37) | 24 (23) | 13 (24) | 27 (25) |
| Non-Hispanic Black | 210 (23) | 79 (29) | 61 (31) | 64 (32) | 40 (38) | 23 (42) | 42 (38) |
| Hispanic | 275 (30) | 62 (23) | 51 (26) | 52 (26) | 34 (33) | 15 (27) | 35 (32) |
| Other | 35 (4) | 11 (4) | 10 (5) | 10 (5) | 6 (6) | 4 (7) | 6 (5) |
| Intensive care during enrollment hospitalization^§^ | 141 (15) | 36 (13) | 22 (11) | 24 (12) | 10 (10) | 6 (11) | 11 (10) |
| *Exposure variables* |  |  |  |  |  |  |  |
| Maternal AD history |  |  |  |  |  |  |  |
| Yes | 117 (13) | 62 (23) | 31 (16) | 34 (17) | 23 (22) | 11 (20) | 25 (23) |
| No | 794 (87) | 205 (77) | 163 (84) | 165 (83) | 81 (78) | 44 (80) | 85 (77) |
| Paternal AD history |  |  |  |  |  |  |  |
| Yes | 73 (8) | 42 (16) | 24 (12) | 26 (13) | 13 (13) | 8 (15) | 14 (13) |
| No | 795 (86) | 208 (78) | 156 (80) | 159 (80) | 81 (78) | 42 (76) | 86 (78) |
| Unknown | 52 (6) | 18 (7) | 15 (8) | 15 (8) | 10 (10) | 5 (9) | 10 (9) |
| Maternal or paternal AD history |  |  |  |  |  |  |  |
| Yes | 175 (19) | 91 (34) | 49 (25) | 54 (27) | 32 (31) | 16 (29) | 34 (31) |
| No | 693 (75) | 162 (60) | 131 (67) | 131 (66) | 63 (61) | 35 (64) | 67 (61) |
| Unknown | 52 (6) | 15 (6) | 15 (8) | 15 (8) | 9 (9) | 4 (7) | 9 (8) |
| Maternal allergic rhinitis history |  |  |  |  |  |  |  |
| Yes | 212 (23) | 89 (33) | 47 (24) | 50 (25) | 24 (23) | 15 (27) | 26 (24) |
| No | 699 (77) | 178 (67) | 147 (76) | 149 (75) | 80 (77) | 40 (73) | 84 (76) |
| Maternal asthma history |  |  |  |  |  |  |  |
| Yes | 195 (21) | 83 (31) | 52 (27) | 55 (28) | 31 (30) | 18 (33) | 33 (30) |
| No | 716 (79) | 184 (69) | 142 (73) | 144 (72) | 73 (70) | 37 (67) | 77 (70) |
| *Child AD definition components* |  |  |  |  |  |  |  |
| Parent-reported AD | 268 (29) | 268 (100) | 131 (67) | 137 (69) | 78 (75) | 47 (85) | 83 (75) |
| AD in medical record | 195 (21) | 131 (49) | 195 (100) | 189 (95) | 99 (95) | 53 (96) | 105 (95) |
| Hybrid AD^†^ | 200 (22) | 137 (51) | 189 (97) | 200 (100) | 104 (100) | 55 (100) | 110 (100) |
| Elevated tIgE | 387 (42) | 131 (49) | 102 (52) | 104 (52) | 104 (100) | 49 (89) | 104 (95) |
| Elevated sIgE^‡^ | 182 (20) | 80 (30) | 56 (29) | 55 (28) | 49 (47) | 55 (100) | 55 (50) |
| Elevated tIgE or sIgE | 415 (45) | 144 (54) | 109 (56) | 110 (55) | 104 (100) | 55 (100) | 110 (100) |

Abbreviations: AD, atopic dermatitis; IQR, interquartile range; sIgE, specific IgE; tIgE, total IgE

^†^ Physician-ascertained AD based on parent report and medical record review

^‡^ Positive results to any food allergen using ImmunoCAP, or positive results to any food or aeroallergen using ISAC

^§^ Intensive care unit, continuous positive airway pressure, and/or intubation

**Supplementary Figure 1.** Overlap between 3 definitions of infant atopic dermatitis

The 3 definitions shown are parent-reported atopic dermatitis, clinician-diagnosed atopic dermatitis ascertained from medical record review, and physician-ascertained atopic dermatitis based on parent report and medical record review (“hybrid” definition).

**Supplementary Figure 2.** Overlap between elevated tIgE and sIgE among infants with atopic dermatitis (“hybrid” definition)

Elevated sIgE includes positive results to any food allergen using ImmunoCAP, or positive results to any food or aeroallergen using Immuno Solid-phase Allergen Chip. “Hybrid” atopic dermatitis refers to physician-ascertained atopic dermatitis based on parent report and medical record review.
