# Supplementary figures and images for: A comparison of case definitions for infant atopic dermatitis in a multicenter prospective cohort study
Source: Health Sci Rep. 2021 Jul 12;4(3):e324. doi: 10.1002/hsr2.324 (PMC8273877; doi:10.1002/hsr2.324)

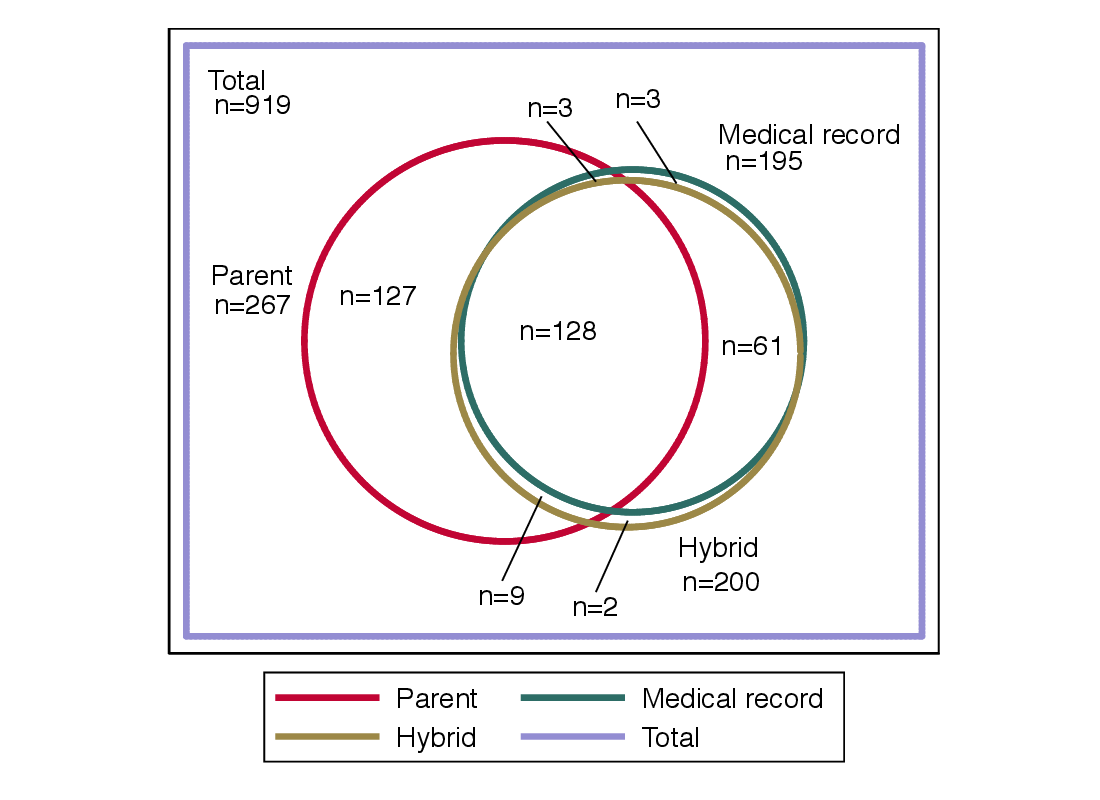

Supplement: Supplementary file 2 — Figure S1. Overlap between three definitions of infant atopic dermatitis The three definitions shown are parent‐reported atopic dermatitis, clinician‐diagnosed atopic dermatitis ascertained from medical record review and physician‐ascertained atopic dermatitis based on parent report and medical record review (“hybrid” definition). [file HSR2-4-e324-s003.tiff]

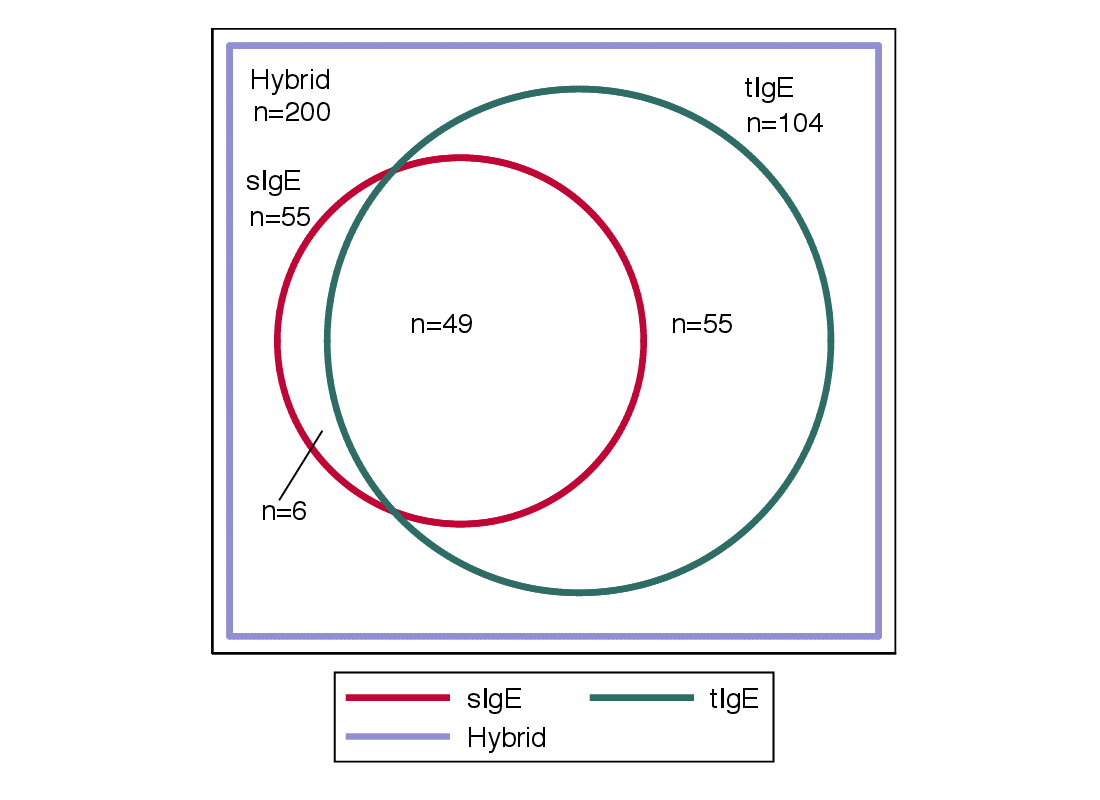

Supplement: Supplementary file 3 — Figure S2. Overlap between elevated total IgE (tIgE) and specific IgE (sIgE) among infants with atopic dermatitis (“hybrid” definition) Elevated sIgE includes positive results to any food allergen using ImmunoCAP or positive results to any food or aeroallergen using Immuno Solid‐phase Allergen Chip. “Hybrid” atopic dermatitis refers to physician‐ascertained atopic dermatitis based on parent report and medical record review. IgE, immunoglobulin E [file HSR2-4-e324-s002.tiff]
